# A cross sectional assessment of basic needs insecurity prevalence and associated factors among college students enrolled at a large, public university in the Southeastern U.S

**DOI:** 10.1186/s12889-022-12817-6

**Published:** 2022-03-02

**Authors:** Mary Kate Robbins, Marsha Spence, Elizabeth Anderson Steeves

**Affiliations:** Department of Nutrition, University of Tennessee, 1215 W. Cumberland Ave. Room229 Jessie Harris Building, Knoxville, TN 37996-1920 USA

**Keywords:** Food insecurity, Housing insecurity, Basic needs insecurity, College, University, Food security status, Hunger, Student, Hunger vital sign

## Abstract

**Background:**

There is increasing evidence of problematic rates of food insecurity among college students, yet few studies have gone beyond this to examine housing insecurity rates or rates of basic need insecurity (BNI), which is defined as having both food and housing insecurity, among the postsecondary population. BNI may have significant impacts on the mental and social health, and academic outcomes of college students, yet remains understudied. The researchers of this study are among the first to assess the prevalence of food insecurity, housing insecurity, and basic needs insecurity among college students enrolled at a large, public university in the Southeast and to identify factors associated with experiencing food, housing, and basic needs insecurity.

**Methods:**

A cross-sectional online survey was conducted at a large, public university in the Southeast United States. All eligible, enrolled students (*n* = 23,444) were asked to complete an online survey, 2634 responded (11.2% response rate). Multivariate logistic regression models were used to assess relationships between demographic and financial factors and the outcomes of interest (food, housing, and basic needs insecurity).

**Results:**

High rates of food insecurity (48.5%), housing insecurity (66.1%), and basic needs insecurity (37.1%) were identified. After controlling for confounders, factors that were significantly associated with increased odds of students having basic needs insecurity included previous food insecurity (*p* < 0.001; Odds Ratio (OR) = 3.36; Confidence Interval (CI) = 2.64–4.28), being employed (*p* < 0.001, OR = 1.70; CI = 1.34–2.17), not receiving family financial support (*p* < 0.001, OR = 1.61; CI = 1.30–2.00), and living off-campus (*p* < 0.001, OR = 1.67; CI = 1.25–2.22). Juniors (*p* < 0.001, OR = 1.78; CI = 1.31–2.42), seniors (*p* < 0.001, OR = 2.06; CI = 1.52–2.78), Masters (*p* = 0.004, OR = 1.68; CI = 1.18–2.40), and PhD or EdD (*p* = 0.029, OR = 1.55; CI = 1.05–2.31) students were significantly more likely to experience basic needs insecurity than sophomore students.

**Conclusions:**

This research identifies high rates of food, housing, and basic needs insecurity among college students enrolled at a large, public university. Financial factors such as being food insecure prior to attending college, working during college, and not having familial financial support were all related to BNI in this sample. Students who were more advanced in their education experienced more BNI than less advanced students. Innovative interventions with enhanced BNI measures, for example, partnering with financial aid offices to screen and refer students to food resources, are likely needed to address this multi-faceted problem.

**Supplementary Information:**

The online version contains supplementary material available at 10.1186/s12889-022-12817-6.

## Background

Basic needs insecurity (BNI) includes insecurity or instability related to food, shelter, water and safety [1]. According to Maslow’s Hierarchy of Needs theory, psychological and self-fulfillment needs cannot be met if basic needs, such as the need for nutritious foods and safe housing, are not met [2]. Applying this theory to a college or university setting, students experiencing elements of BNI have been reported to have difficulty concentrating and completing courses and degree tracks [3]. Given that scholarly and creative work is often considered hallmarks of the college experience, BNI could be particularly problematic for college students. As students apply themselves to their studies, focus on academic pursuits, and the social involvement of attending higher education, BNI may be a barrier to a successful academic experience and healthy social interactions [3, 4]. The small amount of published qualitative research that contextualizes some of the lived experiences of students with food insecurity and housing insecurity, emphasizes that these students report feelings of stigma and shame, as well as the need for to make tradeoffs between focusing on their basic needs such as having enough food and secure housing and their academic priorities [4–6], such as attending classes and purchasing textbooks.

There is some evidence that BNI is problematic among college students [3, 7]. While this is an emerging issue in the grey literature, it is almost entirely lacking in the peer-reviewed literature, which has focused instead on the components of BNI, such as food insecurity (FI) [5–7] and housing insecurity (HI) [1, 7–12]. Given the health and economic impacts of BNI, additional peer-reviewed assessments are warranted [1].

Among college students, BNI is measured through assessments of FI and HI [13]. The U.S. Department of Agriculture (USDA) defines FI as “the lack of consistent access to enough food for an active, healthy life.” [14] HI is identified by the U.S. Department of Health and Human Services using five signs: high housing costs relative to income (greater than 50%), low housing quality (insufficient plumbing, heat, electricity, leaks, or holes), neighborhood instability (high rates of poverty, crime, and unemployment; and poor city services such as litter, noise, and pollution), overcrowding, and the condition of homelessness as the most severe form of HI [15]. College students have not traditionally been included in FI and HI assessments, until recently [3, 7].

Only two peer-reviewed studies have assessed HI and FI, both finding that around 43–44% of students were food insecure [3, 7], and 8% [3] and 52% [7] of students as housing insecure. Yet neither of these studies analyze the combined impact of being both food and housing insecure. There are also a handful of campus community assessments reporting both FI and HI. In these five reports, the rate of FI ranged from 39 to 48% [1, 10, 12, 16, 17]. Three of the five reports from the grey literature reported rates of HI ranging from 43 to 48%, by implementing multiple questions to assess HI [1, 11, 12]. Two of the five reports only assessed homelessness and not HI, with 5 and 11% of surveyed students reporting experiences of homelessness in the past year [10, 17]. One report in the grey literature included measures of BNI, or students that were experiencing both FI and HI. This report found that 30% of students were basic needs insecure, or both food and housing insecure, compared to 39% of students experiencing no insecurities or were basic needs secure [1]. All of these reports provides valuable but difficult to interpret information given inconsistencies in both FI and HI measures used, in addition, only one assesses the prevalence of BNI, instead of the individual components of FI or HI.

Of the components of BNI, FI among college students has been more commonly documented in the peer-reviewed literature. Two systematic reviews of studies assessing college FI found rates of 42% [8] and 43.5% [9], while a recent review estimated a rate of 41% [18]. Nikolaus and colleagues (2020) investigated quality of college FI studies, indicating that the use of differing assessment protocols produces a large range of results across institutions [18]. While there is a lack of peer-reviewed literature in relation to BNI, we can look to these previous studies of FI to start to identify factors that could also be influencing basic need insecurity status. Studies have found that student FI is associated with identifying as a minority race/ethnicity [1, 10, 12, 16, 17], being a member of the LGBTQIA (lesbian, gay, bisexual, transgender, queer/questioning, intersex, asexual) community [1], a former foster youth [1], a first-generation college student [12, 17], and having previous experiences with FI [3, 10]. Characteristics such as being a Pell grant recipient [1, 3, 17], transfer student [17], a student supporting themself financially [1, 16], working over 20 h per week [1, 3, 12, 16], or participating in a federal work study program [17] were associated with FI. While the literature on housing insecurity is much more limited, Tsui and colleagues (2011) found that HI was more likely for students with children than without, and women compared to men [11].

A national report surveying university Presidents, demonstrated that student health and wellness has become a major concern in relation to retention and the general well-being of students compared to previous years with 82% of public, four-year university presidents (*n* = 400) indicating reallocating or finding additional funding for these issues [19]. As issues related to student well-being are becoming more recognized across the nation, assessing BNI will help colleges and universities understand the extent of this issue. There have only been two peer-reviewed studies to assess to assess both FI and HI [3, 7], but neither of these previous studies included the combined variable of BNI. To the research team’s knowledge, this is the first peer-reviewed study to report on rates of, and factors related to BNI at a large land-grant, public university in the Southeast. Southeastern states consistently rank among the worst for health in the U.S. [20], making research in this region particularly important. Exploratory peer-reviewed research is needed to understand BNI and identify the factors that are associated with an increased likelihood of experiencing BNI. Understanding and addressing factors related to these experiences will strengthen the evidence in this area and will allow university administrators to make better, more informed decisions about how to address these issues in on their campuses. This research used cross sectional survey data to 1) assess the prevalence BNI, defined using recommended measurement protocols [13] as the percent of students experiencing both FI and HI, among students attending a large, public university in the Southeast and 2) identify factors that are associated with experiencing student BNI.

## Methods

### Study design and survey development

This study used an online cross-sectional survey, which aimed to assess FI, HI, and BNI status and associated factors. Cross-sectional study designs are often used to measure the prevalence of health-related issues/outcomes and related factors and can contribute significantly to the literature when used for establishing preliminary evidence in a certain topic area [21], as is the case with BNI in this study. The survey was developed by the Southeastern University Consortium on Hunger, Poverty, and Nutrition [22]. The Southeastern University Consortium on Hunger, Poverty, and Nutrition is a partnership of researchers from universities across the Southeastern US, as well as food assistance program professionals and obesity and chronic disease prevention specialists [22] with a shared goal of reducing FI and reducing health disparities [22].

### Setting - population, participants and sampling

This study conducted a census of the entire student population [23], as all students enrolled in October 2019 (total *n* = 29,871) at a large, public university in the Southeast were invited to participate in this survey. The survey was conducted over 5 weeks in October–November 2019 through a series of three emails containing an anonymous survey link (one initial invitation to participate, and two follow up emails) to an all-campus listserv. Freshman students (*n* = 6427) were included on the listserv but were excluded from the analyses, because freshman students.

had only been on campus for a few weeks at the time of the survey. Removal of freshman made the total possible *n* = 23,444 (sophomores, juniors, seniors, graduate, and professional students). There were 2634 total survey respondents for a response rate of 11.2%. Responses with incomplete BNI data were dropped (*n* = 120), creating a sample size of 2514 (10.7% response rate).

Sociodemographic information on the student population at this university (as compared to the sample) can be seen in Table 1. After completing the survey, participants had the opportunity to enter a drawing to win one of ten $50 Amazon e-gift cards by entering their email into a separate survey linked to the end of the data collection survey. All participants were provided an informed consent statement and agreed to participate. This study was reviewed and deemed exempt by the Institutional Review Board at the University (IRB-19-05519-XM).

**Table 1:** Characteristics of the University Population and Study Sample (*n* = 2514)^a^, 2019

Variable	University Population n (column %)	Total Sample n (column %)	Food Insecure n (column %)	Housing Insecure n (column %)	Basic Needs Insecure n (column %)
Gender (*n* = 2510)					
*Female*	15,272 (51.8)	1754 (69.8)	858 (70.5)	1196 (72.0)	672 (72.3)
*Male*	14,188 (48.2)	720 (28.6)	336 (27.6)	441 (26.6)	241 (25.9)
*Other*	–	36 (1.4)	23 (1.9)	24 (1.4)	17 (1.8)
Race (*n* = 2507)					
*American Indian/Alaskan Native*	55 (0.2)	4 (0.2)	4 (0.3)	1 (.06)	1 (0.1)
*Asian*	1105 (3.9)	174 (6.9)	64 (5.3)	102 (6.1)	46 (5.0)
*Black or African American*	1705 (6.1)	106 (4.2)	71 (5.8)	66 (4.0)	48 (5.2)
*Caucasian or White*	22,575 (80.3)	2082 (82.8)	994 (81.9)	1386 (83.6)	766 (82.6)
*Bi or Multiracial*	1043 (3.7)	97 (3.9)	54 (4.4)	73 (4.4)	45 (4.9)
*Other*	1646 (5.9)	44 (1.8)	27 (2.2)	29 (1.8)	21 (2.3)
Ethnicity (*n* = 2509)					
*Hispanic or Latino*	1331 (4.5)	125 (5.0)	81 (6.7)	82 (4.9)	61 (6.6)
*Not Hispanic or Latino*	28,129 (95.5)	2384 (94.8)	1134 (93.3)	1577 (95.1)	867 (93.4)
Age mean ± SD (*n* = 2512)					
*< 18–20*	13,126 (44.6)	758 (30.2)			
*21–25*	11,526 (39.2)	1130 (44.9)			
*26–30*	2523 (8.6)	339 (13.5)	23.57 ± 5.746	23.98 ± 5.505	23.94 ± 5.621
*31 and over*	2249 (7.6)	285 (11.3)			
Year in school (*n* = 2514)					
*Sophomore*	5015.8 (24.0)	479 (19.1)	241 (19.8)	204 (12.3)	122 (13.1)
*Junior*	5272.2 (25.3)	459 (18.3)	260 (21.3)	304 (18.3)	186 (20.0)
*Senior*	5694.5 (27.3)	595 (23.7)	291 (23.9)	447 (26.9)	247 (26.5)
*Master’s*	3892.5 (18.6)	468 (18.6)	207 (17.0)	321 (19.3)	183 (19.6)
*PhD or EdD*	---^b^	392 (15.6)	161 (13.2)	300 (18.0)	142 (15.2)
First Generation Student Status (*n* = 2513)					
*First Generation student*	__	609 (24.2)	348 (28.6)	427 (25.7)	273 (29.3)
*Not First Generation student*	__	1904 (75.7)	870 (71.4)	1235 (74.3)	658 (70.7)

^a^Item-level sample size varied

^b^University data combined PhD or EdD demographics into Master’s demographic

### Survey components

#### Demographic factors

Demographic information was collected (gender identity, race, ethnicity, marital status, age, previous food security status, year in school, first generation student status, residence type). Participants’ gender identity was collected with three possible responses (‘male,’ ‘female,’ and ‘other’). The race/ethnicity variable was collected using six categories (as seen in Table 1). The marital status variable was collected using five categories (single, live with partner, married, divorced, widowed). Students reported their age in years and previous food security status (food secure/food insecure). Previous food security status was collected through two questions adapted from a previously developed food security status screener [24]. Two statements were used, similarly to previous literature: ‘before I came to college, we (my parent/guardian and/or I) worried whether our food would run out before we had money to buy more’ and ‘before I came to college, the food we (my parent/guardian and/or I) bought just didn’t last and we didn’t have money to get more [25]. Responses were ‘often,’ ‘sometimes,’ and ‘never,’ with an affirmative response to either question indicating the student as previously food insecure. Academic status information collected included the student’s year in school (‘Sophomore,’ ‘Junior,’ ‘Senior,’ ‘Masters,’ ‘PhD or EdD,’ ‘Professional’) and identification as a first-generation college student (yes/no) and residence type (on-campus/off-campus).

#### Financial factors

Financial information was collected through questions about employment status, average monthly income, financial aid status, and family financial support. Employment status had four answer choices (‘unemployed,’ ‘one or more part-time jobs,’ ‘one full-time job,’ or ‘other’) and the average monthly income of students (not the student’s family) was collected by a sliding scale in which students could answer $0–8000 [26]. Students also answered whether or not they receive some type of financial aid [27] (yes/no) or financial support from family (yes/no) [28].

#### Food insecurity

The 2-item Hunger Vital Sign was used to assess food security status [24]. The 2-item Hunger Vital Sign is a validated brief measure to quickly assess food security status and has less participant burden than other validated measures [24]. The Hunger Vital Sign includes two questions: 1) “Within the past 12 months, I worried whether my food would run out before I got money to buy more”, and 2) “Within the past 12 months, the food I bought just didn’t last and I didn’t have money to get more.” The Hunger Vital Sign has been validated and found effective by multiple studies [24, 29], is quick, easy to administer and has a sensitivity of 97%, specificity of 83% [24], and is the recommended food security screening tool by leading medical and anti-hunger organizations [30, 31] has been used previously in the literature with a college students [32].

In this study, the Hunger Vital Sign questions were modified, to begin with “Since starting college,” followed by the question to capture FI status during college rather than collecting data from the standard timeframe used in the survey. Survey respondents could answer ‘often true,’ ‘sometimes true,’ or ‘never true’ to questions asking about frequency of an experience related to FI. An affirmative response to either question classified the respondent as ‘food insecure’, while “never true” responses to both questions classified the respondent as ‘food secure’.

#### Housing insecurity

A 6-item series of questions modified from the national Survey of Income and Program Participation (SIPP) from the United States Census Bureau was used to assess HI status [33]. This 6-item series was recommended for use with college students [13, 17], and used in whole or in part by both of the previous peer-reviewed studies of college housing insecurity [3, 7] and in the grey literature [1]. Survey questions asked about whether or not there was a rent or mortgage increase that made it difficult to pay, if the student did not pay or underpaid their rent or mortgage, if the student did not pay the full amount of a gas, oil, or electricity bill, if the student moved two or more times, if the student moved in with other people, even for a little while, because of financial problems, and if the student lives with others beyond the expected capacity of the house or apartment. All six questions prompted a ‘yes’ or ‘no’ response. When scoring, an affirmative response to any of the six HI questions signified the student as ‘housing insecure’, while negative responses to all six HI questions classified the student as ‘housing secure’ [13, 17].

#### Basic needs insecurity

Those who were both food and housing insecure on the 2-item Hunger Vital Sign and HI questions, respectively, were classified as ‘basic needs insecure.’ The category for ‘basic needs secure’ included all other classifications (‘food secure and housing secure,’ ‘food secure and housing insecure,’ ‘food insecure and housing secure’).

### Data management

Upon completion of data collection, survey data were uploaded to SPSS Software 2.0 (IBM SPSS Statistics for Windows Version 25.0, Armonk, New York) for data cleaning and analysis. The race/ethnicity variable and marital status variable were condensed to dichotomous responses before data analysis due to small sample sizes among certain categories. The race/ethnicity variable was collected using six categories (as seen in Table 1), then condensed to a binary variable of white/non-white for analysis. The marital status variable was collected using five categories (single, live with partner, married, divorced, widowed) and condensed to single/partnered for analysis. Missing data and incomplete responses were examined, and frequencies were conducted to identify the amount of missing data (Additional file 1). Little’s Missing Completely at Random (MCAR) test identified that missing data were missing at random (*p* = .123). Because the missing data were determined to be missing at random, participants with missing data for any given analysis were dropped from the analysis, thus the sample sizes for these analyses vary slightly and are noted in the relevant tables or figures. Outliers were examined to determine if they were true outliers by exploring if the responses are greater or less than three times the interquartile range for each variable [34]. There were numerous true outliers for the students’ monthly income (*n* = 79) variable. True outliers remained in the dataset, while unfounded responses were removed from the dataset. In the case of numerous outliers, sensitivity analyses were conducted and found that the outliers did not influence the data analysis results.

### Statistical analyses

Descriptive statistics were conducted to determine characteristics of the sample and levels of BNI, as well as FI and HI. Chi square tests and independent t-tests were used to determine associations between BNI status, and the variables related to student demographics (gender identity, age, race, ethnicity, year in school, residence type, first generation student status, marital status, previous food security status), financial factors (employment status, student average monthly income, financial aid status/receiving financial aid, family support). Similar analyses were conducted with the other dependent variables, FI and HI.

Next, for the adjusted analyses, three separate logistic regression models were built. One model had basic needs security status (categorized as basic needs secure versus basic needs insecure) as the dependent variable. The second model and third model had food security status (categorized as food secure versus food insecure) and housing security status (categorized as housing secure versus housing insecure), respectively, as the dependent variables. Significant relationships identified in the chi square and t-tests, along with insight from the literature [1, 10–12, 16, 17], were used to inform the variables included in the logistic regression models [35]. The independent variables included student demographics (year in school, marital status, first-generation college student status, previous food security status, on- or off-campus residence) and financial factors (employment status, student average monthly income, family financial support). Confounders were included in the model based on previous literature, including age, race/ethnicity, and gender [26–38]. Each logistic regression model was checked for the assumptions that the variables were independent, the sample size was large enough (400+), and that the dependent variables were binary [39]. Multi-collinearity was assessed, and the financial aid and student monthly income variables were found to have overlap. Both variables were included in versions of the model, but the financial aid variable was not significant, so it was removed in favor of a more parsimonious model. In addition, professional students (dental, law, medical, and nursing students) were removed from the analyses due to small sample size (*n* = 121). The Hosmer Lemeshow goodness of fit test was used with each of the three logistic regression models to assess how well each model predicted the outcomes, and found that the fit of the models was good [39]. Statistical significance for all tests was determined at alpha <.05 level.

## Results

### Sample

Survey respondents were predominately female (60.8%, *n* = 1754), undergraduate (61.1%, *n* = 1533) students (Table 1). Approximately 83% of survey respondents identified as White (*n* = 2082), and 5% of the study sample identified as Hispanic or Latino of any race (*n* = 125). The mean age of survey respondents was 24.0 ± 6.20 years. Nearly one-fourth of the study sample identified as a first-generation college student (*n* = 609). Of the student respondents, 48.5% were classified as food insecure. Nearly 46% reported that since starting college they worried whether food would run out before they got money to buy more, (*n* = 1153), and 31.7% reported that the food they bought just didn’t last and not having the money to get more (*n* = 796). Using the 6-item series of questions to score HI status, 66.1% of the sample (*n* = 2514) were classified as housing insecure. A breakdown of the responses to each HI question can be seen in the Additional file 2.

Among the respondents (*n* = 2514), 37.1% of student were classified as basic needs insecure (both food insecure and housing insecure). Of the students who were not basic needs insecure, 23% were both food and housing secure, 29% were food secure and housing insecure, and 11% were food insecure and housing secure. Of the respondents classified as basic need insecure, the majority self-identified as female (72.3%) and Caucasian or white (82.6%) Approximately 5% of the students classified as having BNI self-identified as Asian (5.0%), Black or African American (5.2%), or multiracial (4.9%), and 6.6% identified as Hispanic (Table 1).

### Individual factors associated with basic needs insecurity, food insecurity, and housing insecurity

Chi square analyses and independent t-tests between factors of interest (food security status before college, on- or off-campus residency, first-generation student status, employment.

status, family financial support, student monthly income) and food, housing and basic need insecurity statuses indicated similar results with some variation. Chi square analyses showed students who were employed (*p* < 0.01), were a first-generation college student (*p* < 0.01), experienced FI before college (*p* < 0.01), and did not receive family financial support (*p* < 0.01).

had significant associations with BNI, FI, and HI. Independent t tests showed students who had lower student monthly income (*p* < 0.01) were also associated with BNI, FI, and HI.

Students who identified as Hispanic or Latino (*p* = 0.01), female (*p* = 0.03), living off-campus (*p* < 0.01), and receiving financial aid (*p* = 0.03) were associated with one or two insecurities (BNI, FI, or HI), but not all three. The variables ‘year in school’ and ‘age’ were significant in the bivariate analyses with one or two insecurities (BNI, FI, or HI), but not all three (Additional files 3, 4, 5).

### Factors associated with BNI, HI, and FI using adjusted multivariate logistic regression models

Three adjusted multivariate logistic regression models were constructed to assess the relationships between dependent factors (employment status, first generation status, previous food security status, family financial support, student monthly income, marital status, residence, year in school) and outcomes of interest BNI, FI, HI.

### Basic needs insecurity

In the BNI model, previous food security status was the strongest correlate of BNI (odds ratio (OR), 3.36; 95% confidence interval (CI), 2.64–4.28), indicating that students with previous FI were 3.36 times more likely to experience BNI than the students who did not experience FI before college. Seniors were 2.06 times (CI, 1.52–2.78) more likely to be basic needs insecure than sophomore students. Students who were employed were 1.70 times more likely to have BNI than students who were not employed (CI, 1.34–2.17). Students not receiving family financial support were 1.61 times more likely to have BNI compared to students receiving family financial support (CI, 1.30–2.00), and BNI was 1.67 times more likely for students who lived off-campus compared to students who lived on-campus (CI, 1.25–2.22). Race, age, gender, ethnicity were controlled for in the model and the Hosmer-Lemeshow test indicated the model was a good fit (χ^2^ = 2.530, *p* = 0.960). Results can be seen in Table 2.

**Table 2:** Multivariate Logistic Regression of Factors Associated with Basic Needs Insecurity, 2019

Variable (reference category^a^)	*B*	*P value*	Odds Ratio	95% CI
Year in school (Sophomore)				
*Junior*	0.58	**< 0.01**	1.78	1.31–2.42
*Senior*	0.72	**< 0.01**	2.06	1.52–2.78
*Masters*	0.52	**< 0.01**	1.68	1.18–2.40
*PhD or EdD*	0.44	**0.03**	1.55	1.05–2.31
Previous Food Security Status (Previously Food Secure)				
*Previously Food Insecure*	1.21	**< 0.01**	3.36	2.64–4.28
Student monthly income	−0.17	**< 0.01**	0.85	0.77–0.92
Family financial support (Yes)				
*No*	0.48	**< 0.01**	1.61	1.30–2.00
Employment status (Employed)				
*Unemployed*	0.53	**< 0.01**	1.70	1.34–2.17
Gender (Male)				
*Female*	0.15	0.16	1.16	0.94–1.42
Race (Caucasian/white)				
*Non-white*	−0.07	0.57	0.93	0.73–1.19
Ethnicity (Non-Hispanic)				
*Hispanic*	0.40	0.06	1.49	0.98–2.25
Marital Status (Single)				
*Partnered*	0.06	0.67	1.06	0.82–1.36
Residence (On-campus)				
*Off-campus*	0.51	**< 0.01**	1.67	1.25–2.22
First Generation Student Status (Yes)				
*No*	−0.04	0.74	0.96	0.77–1.20
Age	−0.03	**0.01**	0.97	0.95–0.99

α, *p* < 0.05, significant values are bolded.

^a^Reference category included for categorical variables

### Food insecurity

Similarly to the BNI model, previous food security status was the strongest correlate in the FI model (CI, 3.68–6.26), with students being 4.80 times more likely to being FI in college if.

the student was previously FI. Also, students who were employed and not receiving family financial support were more likely to have FI. If employed, students were 1.44 times more likely to experience FI compared to those who were unemployed (CI, 1.16–1.80), and those who had not received family financial support were 1.30 times more likely to be food insecure than those who had received familial financial support (CI, 1.05–1.61). Race, age, gender, and ethnicity were controlled for in the model and the Hosmer-Lemeshow test indicated the model was a good fit (χ^2^ = 10.70, *p* = 0.22). Results can be seen in Table 3.

**Table 3:** Multivariate Logistic Regression of Factors Associated with Food Insecurity, 2019

Variable (reference category^a^)	*B*	*P value*	Odds Ratio	95% CI
Year in school (Sophomore)				
*Junior*	0.18	0.23	1.19	0.89–1.59
*Senior*	−0.01	0.94	0.99	0.75–1.31
*Masters*	−0.27	0.11	0.76	0.54–1.07
*PhD or EdD*	−0.36	0.06	0.69	0.48–1.01
Previous Food Security Status (Previously Food Secure)				
*Previously Food Insecure*	1.57	**< 0.01**	4.79	3.68–6.26
Student monthly income	−0.11	**< 0.01**	0.89	0.83–0.96
Family financial support (Yes)				
*No*	0.26	**0.02**	1.30	1.05–1.61
Employment status (Employed)				
*Unemployed*	0.37	**0.01**	1.443	1.16–1.80
Gender (Male)				
*Female*	0.03	0.80	1.03	0.85–1.25
Race (Caucasian/white)				
*Non-white*	−0.10	0.44	0.91	0.72–1.16
Ethnicity (Non-Hispanic or Latino)				
*Hispanic*	0.51	**0.02**	1.66	1.08–2.53
Marital Status (Single)				
*Partnered*	−0.05	0.72	0.96	0.74–1.23
Residence (On-campus)				
*Off-campus*	−0.08	0.55	0.92	0.71–1.20
First Generation Student Status (Yes)				
*No*	−0.10	0.39	0.91	0.73–1.13
Age	−.018	0.09	0.98	0.96–1.00

α, *p* < 0.05, significant values are bolded.

^a^Reference category included for categorical variables

### Housing insecurity

Previous food security status was again a strong correlate of HI (CI, 1.53–2.68), indicating that students with previous FI were twice as likely to be housing insecure than the students who did not experience FI before college. Being employed and not receiving family financial support increased the likelihood of being housing insecure. Employed students were 1.53 times more likely to experience HI (CI, 1.22–1.92), and students not receiving family financial support were 1.70 times more likely to experience HI if not receiving family financial support (CI, 1.34–2.15).

In the HI model, students who identified as female were 1.36 times more likely to be housing insecure compared to students who identified as male (CI, 1.11–1.67). Last, using sophomores as the reference group, juniors, seniors, Master’s, or PhD or EdD students were.

significantly more likely to have HI. Notably, seniors were 4.45 times (CI, 3.31–5.97) more likely and PhD or EdD students were 6.25 times (CI, 4.16–9.39) more likely to be housing insecure than sophomores. Race, age, gender, and ethnicity were controlled for in the model and the Hosmer-Lemeshow test indicated the model was a good fit (χ^2^ = 9.349, *p* = 0.314). Results can be seen in Table 4.

**Table 4:** Multivariate Logistic Regression of Factors Associated with Housing Insecurity, 2019

Variable (reference category^a^)	*B*	*P value*	Odds Ratio	95% CI
Year in school (Sophomore)				
*Junior*	0.91	**< 0.01**	2.48	1.85–3.31
*Senior*	1.49	**< 0.01**	4.45	3.31–5.97
*Masters*	1.24	**< 0.01**	3.44	2.42–4.88
*PhD or EdD*	1.83	**< 0.01**	6.25	4.16–9.39
Previous Food Security Status (Previously Food Secure)				
*Previously Food Insecure*	0.71	**< 0.01**	2.03	1.53–2.68
Student monthly income	−0.14	**< 0.01**	0.87	0.80–0.93
Family financial support (Yes)				
*No*	0.53	**< 0.01**	1.70	1.34–2.15
Employment status (Employed)				
*Unemployed*	0.42	**< 0.01**	1.53	1.22–1.92
Gender (Male)				
*Female*	.31	**< 0.01**	1.36	1.11–1.67
Race (Caucasian/white)				
*Non-white*	0.10	0.46	1.10	0.85–1.42
Ethnicity (Non-Hispanic or Latino)				
*Hispanic*	−0.06	0.79	0.94	0.61–1.46
Marital Status (Single)				
*Partnered*	0.30	**0.04**	1.35	1.02–1.79
Residence (On-campus)				
*Off-campus*	0.68	**< 0.01**	1.98	1.52–2.57
First Generation Student Status (Yes)				
*No*	−0.01	0.95	0.99	0.78–1.26
Age	−0.08	**< 0.01**	0.93	0.91–0.95

α, *p* < 0.05, significant values are bolded.

^a^Reference category included for categorical variables

## Discussion

This is one of the first studies to assess the prevalence of BNI among college students attending a large, public university in the Southeast and to identify factors that are associated with experiencing student BNI, and the first study to examine the dual impact of FI and HI by additionally conducting analyses by BNI status [3, 7]. One peer-reviewed study measured FI and HI, finding that 8.0% of students were both food and housing insecure [3], while one non-peer reviewed report included measures of BNI, finding that 30.0% of students were both food and housing insecure [1]; this study found a slightly higher rate of BNI (37.1%). Further, this study found a higher rate of HI (66.1%) than the rates of student HI in previous reports, which ranged from 8.0–48% [1, 11, 12]. In previous reports, the prevalence of FI ranged from 39 to 48% [1, 10, 12, 16, 17], while systematic reviews of studies assessing college FI found rates of 42% [8], 43.5% [9], and 41% [18]. The prevalence of FI in this study (48.5%) was similar to one report [38], but slightly higher than the average. However, comparisons of rates across studies can be difficult because there is inconsistency in the tools being used to measure FI across studies, which likely impacts the results. Thus, the differences seen across studies could be due to differences in tools used to measure FI as well as different reference periods [18]. The same reasoning applies to HI. One peer-reviewed study and one national report used the exact same questions as this study and found that 52 and 56% of students experienced HI, respectively [1, 7], while this study found 66.1% had HI. While this study used the recommended and most widely used tool to assess HI among college students there is not a validated measure for HI, and thus, since HI is a sub-component of BNI, there is also not validated measure for BNI, increasing the difficulty in comparing results across studies. The development of a validated measure for HI and consistent use of the measure is critical to moving this field forward. In addition to development of better tools to assess HI among college students, more studies of nationally representative samples that consistently use both FI and HI measures would contribute to better comparisons in this area of study. The National College Health Association has recognized this need and will start including more questions on food and housing insecurity in the 2022 National College Health Assessment Survey [40].

Results from the multivariate regression analyses indicated that students who had experienced FI before coming to college were 3.36 times more likely be basic needs insecure. This is similar to the findings of another study focused solely on FI in the Southeastern region [25], thus this paper expands on the previous literature. Taken together, these findings imply that high rates of BNI among college students may be due in part to FI among students before entering college. University administrators may have limited ability to address structural issues before college but should be aware of this association. Access to higher education has increased through educational programs and initiatives providing high school graduates with opportunities to attend 2- or 4-year colleges or universities with little to no tuition and mandatory fees. As student populations shift towards more non-traditional students [41], who may have more factors associated with BNI than traditional college students, universities should be aware of the relationship with BNI and allocate funding for systematic assessments of BNI and resources to assist students experiencing this issue. Students who were employed or did not receive family financial support were also more likely to experience BNI. While BNI is an emerging focus in the college population, employment [20, 21] and not receiving family financial support [27, 42] have been previously associated with experiencing FI in the college population. Juniors, seniors, masters, and doctoral students were more likely to be housing insecure than sophomores, with seniors and doctoral students having the highest risk. Students living off-campus were more likely to experience BNI. Living on-campus may serve as a protective factor to students, as students who live in campus housing may be more likely to have a campus-sponsored meal plan, although it is important to note that freshmen were excluded from this study. Employment, lack of family financial support, and longer length of time in a degree program may indicate that as students transition to becoming more independent, they may be more susceptible to BNI. Specifically, doctoral students were 6.5 times more likely to experience housing insecurity compared to sophomores, which was similar to the findings of another study [7]. This finding needs to be explored further to determine if there is a unique issue related to housing insecurity that doctoral students are particularly susceptible to, indicating specific interventions needed to address this group. These are factors to be aware of as university administrators address issues of FI and HI. Informed administrators have the ability to create more strategic interventions based upon best practices identified in the literature, such as campus food pantries, housing assistance programs, financial aid screening and refer programs, and financial literacy and budgeting workshops, for students as they become more independent and transition towards adulthood.

This study is novel as BNI among college students in an emerging area of research with little published in the peer-reviewed literature. Additionally, the grey literature on this topic identifies FI and HI prevalence among college students in the Northeast [11, 16], on the West coast [10, 17], and nationally [1, 12]. This study fills a gap by supplementing the research from a large, public university in the Southeast [3], which is a region that consistently ranks among the worst for nutrition-related outcomes in the U.S. [20]

FI has been studied more than HI and has validated measurement tools such as the Hunger Vital Sign used in this study. HI assessment lacks validated measurement tools, which makes it difficult to assess. This study measured HI to be higher than previous reports of HI but is difficult to compare because of the use of different assessment tools. In addition, recall and social desirability biases may be present as the study used a self-report survey data, although this is consistent in the literature related to college FI [8, 9].

A strength of the study included the ability to conduct a census and systematically invite all enrolled students to participate in the survey, however, not all students responded. A response rate of 11.2% was achieved, which is higher than the average response rate for web-based surveys [43]. While the response rate is better than average and a large sample of students completed the survey, there is still a large portion of the student population that did not respond to the survey and therefore, it is uncertain whether students who did not respond to the survey differ from those that did respond. The sample that completed this survey contained more white, female, older, graduate students than the overall population at the university (Table 1). Because the potential for non-response bias is inherent in cross-sectional studies [21], we cannot conclude that the students who responded to this survey accurately represent the overall student population at the university. In addition, while the percentage of the population that responded was fairly small compared to the total population, the analytic sample for this study was quite large and may have the ability to introduce type 1 error, thus, these findings should be interpreted with this limitation in mind.

## Conclusion

Students from this large, public university in the Southeast reported problematic rates of BNI, FI, and HI. College BNI is an emerging field and an important area of future study. A validated measurement tool for HI and consistent use is needed to advance this field, along with more consistent use of FI measurement tools. In addition, accurate information on the financial situation of students is challenging to capture given varying levels of financial independence [25]. More specific questions related to student finances would allow for better understanding of these relationships. Cross-sectional studies are key to understanding prevalence of emerging health and social concerns, such as BNI among college students [2]. However, future research should move beyond cross-sectional study designs and include longitudinal or cohort studies of college BNI, FI, and HI to more dynamically understand the many demographic, social, and financial factors associated with BNI, FI, and HI. These study designs would allow a better understanding of the directionality of the relationships between BNI, FI, and/or HI and related factors, which is missing in the current literature. Additionally, comparisons across different types of schools, such as large, public and small, private colleges and universities, and universities from different geographical regions, and studies including all levels of students (i.e. Freshman) may be one way to determine if some students or schools are at higher risk and will allow measures to be put in place that mitigate that risk. Future studies should also investigate systematic interventions and programming that may alleviate experiences of BNI while in college. This exploratory study, and future studies to further refine our understanding of this issue and potential solutions, can drive critical institutional, state, and federal-level policy supports to assure that diverse and non-traditional postsecondary student populations can not only access higher education, but thrive in that environment.

## Supplementary Information

**Additional file 1.** Frequency of Missing Data and Chi-square Analyses of Missing Data versus Variables of Interest^a^. Table showing the frequencies of missing data for each variable with more than 4% missing data and results of the chi-square analysis of the variable with missing data verses the variables of interest (food security status, housing security status, and basic needs security status).

**Additional file 2. **Reported Housing Security & Insecurity by SIPP Question [32] (*n* = 2514), 2019. Figure depicting the percentage of “yes” and “no” responses to the six SIPP questions used to measure housing security status.

**Additional file 3.** Bivariate Analyses of Basic Needs Security Status with Demographic, Financial, and Academic Factors, 2019. Table showing results of chi-square and independent t tests for the demographic, financial, and academic factors verses the variable of interest (basic needs security status).

**Additional file 4.** Bivariate Analyses of Food Security Status with Demographic, Financial, and Academic Factors, 2019. Table showing results of chi-square and independent t tests for the demographic, financial, and academic factors verses the variable of interest (food security status).

**Additional file 5.** Bivariate Analyses of Housing Security Status with Demographic, Financial, and Academic Factors, 2019. Table showing results of chi-square and independent t tests for the demographic, financial, and academic factors verses the variable of interest (housing security status).

## Data Availability

The datasets generated and/or analyzed during the current study are not publicly available due to ongoing analysis of this data set by the research team. Data can be made available from the corresponding author on reasonable request.

## Abbreviations

BNI: Basic needs insecurity
FI: Food insecurity
HI: Housing insecurity
OR: Odds Ratio
CI: Confidence Interval
USDA: United States Department of Agriculture
SIPP: Survey of Income and Program Participation
MCAR: Little’s Missing Completely at Random
