# Supplementary material for: A cross sectional assessment of basic needs insecurity prevalence and associated factors among college students enrolled at a large, public university in the Southeastern U.S
Source: BMC Public Health. 2022 Mar 2;22:419. doi: 10.1186/s12889-022-12817-6 (PMC8889695; doi:10.1186/s12889-022-12817-6)
Supplement: Supplementary file 1 — Additional file 1. Frequency of Missing Data and Chi-square Analyses of Missing Data versus Variables of Interesta. Table showing the frequencies of missing data for each variable with more than 4% missing data and results of the chi-square analysis of the variable with missing data verses the variables of interest (food security status, housing security status, and basic needs security status). [file 12889_2022_12817_MOESM1_ESM.docx]

Additional File 2. Frequency of Missing Data and Chi-square Analyses of Missing Data versus Variables of Interest^a^

| Variable | Missing Data  n (%) | Food Security Status | Housing Security Status | Basic Needs Security Status |
| --- | --- | --- | --- | --- |
| Current Health | **135 (5.7)** | χ^2^ = 0.07  p = 0.80 | χ^2^ = 0.65  p = 0.42 | χ^2^ = 1.67  p = 0.20 |
| BMI | **200 (8.6)** | χ^2^ = 0.54  p = 0.46 | χ^2^ = 0.26  p = 0.61 | χ^2^ = 3.43  p = 0.06 |
| Poor physical health days | **148 (6.3)** | χ^2^ = 0.02  p = 0.90 | χ^2^ = 0.03  p = 0.87 | χ^2^ = 1.06  p = 0.30 |
| Poor mental health days | **164 (7.0)** | χ^2^ = 0.32  p = 0.57 | χ^2^ = 0.01  p = 0.93 | χ^2^ = 1.70  p = 0.19 |
| Poor usual activities days | **161 (6.8)** | χ^2^ < 0.01  p = 0.99 | χ^2^ = 0.19  p = 0.67 | χ^2^ = 2.15  p = 0.14 |

α, *p*<0.05, significant values are bolded

^a^All other variables of interest had less than 4% missing data
