# Supplementary material for: A cross sectional assessment of basic needs insecurity prevalence and associated factors among college students enrolled at a large, public university in the Southeastern U.S
Source: BMC Public Health. 2022 Mar 2;22:419. doi: 10.1186/s12889-022-12817-6 (PMC8889695; doi:10.1186/s12889-022-12817-6)
Supplement: Supplementary file 2 — Additional file 2. Reported Housing Security & Insecurity by SIPP Question [32] (n = 2514), 2019. Figure depicting the percentage of “yes” and “no” responses to the six SIPP questions used to measure housing security status. [file 12889_2022_12817_MOESM2_ESM.docx]

Additional File Figure 1. Reported Housing Security & Insecurity by SIPP Question^33^ (n=2,514), 2019
