# Supplementary material for: A cross sectional assessment of basic needs insecurity prevalence and associated factors among college students enrolled at a large, public university in the Southeastern U.S
Source: BMC Public Health. 2022 Mar 2;22:419. doi: 10.1186/s12889-022-12817-6 (PMC8889695; doi:10.1186/s12889-022-12817-6)
Supplement: Supplementary file 3 — Additional file 3. Bivariate Analyses of Basic Needs Security Status with Demographic, Financial, and Academic Factors, 2019. Table showing results of chi-square and independent t tests for the demographic, financial, and academic factors verses the variable of interest (basic needs security status). [file 12889_2022_12817_MOESM3_ESM.docx]

Additional File 3. Bivariate Analyses of Basic Needs Security Status with Demographic, Financial, and Academic Factors, 2019

| Variable | Sample  n (%) | Basic Needs Secure  n (%) | Basic Needs Insecure  n (%) | χ^2^ | p |
| --- | --- | --- | --- | --- | --- |
| Current Health  *Excellent/Good*  *Fair/Poor* | 1794 (71.4)  585 (23.3) | 1216 (81.6)  274 (18.4) | 578 (65.0)  311 (35.0) | **82.68** | **<0.01** |
| Employed  *Yes*  *No* | 1857 (73.9)  619 (24.6) | 1114 (70.5)  448 (28.4) | 743 (79.8)  171 (18.4) | **32.54** | **<0.01** |
| Ethnicity  *Hispanic*  *Non-Hispanic* | 125 (5.0)  2384 (94.8) | 64 (4.0)  1517 (96.0) | 61 (6.6)  867 (93.4) | **7.88** | **0.01** |
| First Generation  *Yes*  *No* | 609 (24.2)  1904 (75.7) | 336 (21.2)  1246 (78.8) | 273 (29.3)  658 (70.7) | **20.86** | **<0.01** |
| Food insecure before college  *Yes*  *No* | 469 (18.7)  2043 (81.3) | 175 (11.1)  1405 (88.9) | 294 (31.5)  638 (68.5) | **161.75** | **<0.01** |
| Family financial support  *Yes*  *No* | 1617 (64.3)  880 (35.0) | 1100 (70.2)  468 (29.8) | 517 (55.7)  412 (44.3) | **53.76** | **<0.01** |
| Year in school  *Sophomore*  *Junior*  *Senior*  *Masters*  *PhD or EdD*  *Professional school* | 479 (19.1)  459 (18.3)  595 (23.7)  468 (18.6)  392 (15.6)  121 (4.8) | 357 (22.6)  273 (17.3)  348 (22.0)  285 (18.0)  250 (15.8)  69 (4.4) | 122 (13.1)  186 (20.0)  247 (26.5)  183 (19.6)  142 (15.2)  52 (5.6) | **37.77** | **<0.01** |
| Gender identity  *Male*  *Female*  *Other* | 720 (28.6)  1754 (69.8)  36 (1.4) | 479 (30.3)  1082 (68.5)  19 (1.2) | 241 (25.9)  672 (72.3)  17 (1.8) | **6.75** | **0.03** |
| Race  *White*  *Non-White* | 2082 (82.8)  425 (16.9) | 1316 (83.3)  264 (16.7) | 766 (82.8)  161 (17.4) | 0.18 | 0.67 |
| Residency  *On campus*  *Off campus* | 385 (15.3)  2128 (84.6) | 285 (18.0)  1297 (82.0) | 100 (10.7)  831 (89.3) | **23.90** | **<0.01** |
| Financial Aid  *Yes*  *No* | 1890 (75.2)  621 (24.7) | 1167 (73.9)  413 (26.1) | 723 (77.7)  208 (22.3) | **4.54** | **0.03** |
| Marital status  *Single*  *Partnered* | 1929 (76.7)  584 (23.2) | 1229 (77.7)  352 (22.3) | 700 (75.1)  232 (24.9) | 2.27 | 0.13 |
| BMI  *≤ 21.49*  *21.50-23.89*  *23.90-27.49*  $\boldsymbol{\geq}$*27.50* | 607 (26.2)  560 (24.2)  589 (25.5)  558 (24.1) | 386 (26.7)  357 (24.7)  375 (26.0)  326 (22.6) | 221 (25.4)  203 (23.3)  214 (24.6)  232 (26.7) | 4.97 | 0.17 |
| Poor physical health days  0 days  1-3 days  4-9 days  10-30 days  Don’t know | 985 (39.2)  375 (14.9)  355 (14.1)  247 (9.8)  404 (16.1) | 720 (48.6)  222 (15.0)  189 (12.7)  128 (8.6)  224 (15.1) | 265 (30.0)  153 (17.3)  166 (18.8)  119 (13.5)  180 (20.4) | **82.64** | **<0.01** |
| Poor mental health days  0 days  1-3 days  4-9 days  10-30 days  Don’t know | 482 (19.2)  317 (12.6)  439 (17.5)  858 (34.1)  254 (10.1) | 380 (25.8)  234 (15.9)  287 (19.5)  407 (27.7)  163 (11.1) | 102 (11.6)  83 (9.4)  152 (17.3)  451 (51.3)  91 (10.4) | **157.30** | **<0.01** |
| Poor usual activities days  0 days  1-3 days  4-9 days  10-30 days  Don’t know | 893 (35.5)  412 (16.4)  370 (14.7)  396 (15.8)  282 (11.2) | 676 (45.9)  269 (18.3)  210 (14.3)  162 (11.0)  155 (10.5) | 217 (24.6)  143 (16.2)  160 (18.2)  234 (26.6)  127 (14.4) | **158.66** | **<0.01** |
| Student monthly income | 1094.26 ± 1384.27 | 1164.67 ± 1534.58 | 977.51 ± 1081.06 | - | **<0.01** |

α, *p*<0.05, significant values are bolded
