# Supplementary material for: A cross sectional assessment of basic needs insecurity prevalence and associated factors among college students enrolled at a large, public university in the Southeastern U.S
Source: BMC Public Health. 2022 Mar 2;22:419. doi: 10.1186/s12889-022-12817-6 (PMC8889695; doi:10.1186/s12889-022-12817-6)
Supplement: Supplementary file 4 — Additional file 4. Bivariate Analyses of Food Security Status with Demographic, Financial, and Academic Factors, 2019. Table showing results of chi-square and independent t tests for the demographic, financial, and academic factors verses the variable of interest (food security status). [file 12889_2022_12817_MOESM4_ESM.docx]

Additional File 4. Bivariate Analyses of Food Security Status with Demographic, Financial, and Academic Factors, 2019

| Variable | Sample  n (%) | Food Secure  n (%) | Food Insecure  n (%) | χ^2^ | p |
| --- | --- | --- | --- | --- | --- |
| Housing Security Status  Housing Secure  Housing Insecure | 851 (33.9)  1663 (66.1) | 564 (43.6)  731 (56.4) | 287 (23.5)  932 (76.5) | **112.26** | **<0.01** |
| Current Health  *Excellent/Good*  *Fair/Poor* | 1794 (71.4)  585 (23.3) | 1012 (82.7)  212 (17.3) | 782 (67.7)  373 (32.3) | **71.86** | **<0.01** |
| Employed  *Yes*  *No* | 1857 (73.9)  619 (24.6) | 934 (72.2)  348 (26.9) | 923 (75.8)  271 (22.3) | **10.75** | **0.01** |
| Ethnicity  *Hispanic*  *Non-Hispanic* | 125 (5.0)  2384 (94.8) | 44 (3.4)  1250 (96.6) | 81 (6.7)  1134 (93.3) | **14.12** | **<0.01** |
| First Generation  *Yes*  *No* | 609 (24.2)  1904 (75.7) | 261 (20.2)  1034 (79.8) | 348 (28.6)  870 (71.4) | **24.22** | **<0.01** |
| Food insecure before college  *Yes*  *No* | 469 (18.7)  2043 (81.3) | 97 (7.5)  1197 (92.5) | 372 (30.5)  846 (69.5) | **219.45** | **<0.01** |
| Family financial support  *Yes*  *No* | 1617 (64.3)  880 (35.0) | 879 (68.4)  406 (31.6) | 738 (60.9)  474 (39.1) | **15.43** | **<0.01** |
| Year in school  *Sophomore*  *Junior*  *Senior*  *Masters*  *PhD or EdD*  *Professional school* | 479 (19.1)  459 (18.3)  595 (23.7)  468 (18.6)  392 (15.6)  121 (4.8) | 238 (18.4)  199 (15.4)  304 (23.5)  261 (20.2)  231 (17.8)  62 (4.8) | 241 (19.8)  260 (21.3)  291 (23.9)  207 (17.0)  161 (13.2)  59 (4.8) | **24.94** | **<0.01** |
| Gender identity  *Male*  *Female*  *Other* | 720 (28.6)  1754 (69.8)  36 (1.4) | 384 (29.7)  896 (69.3)  13 (1.0) | 336 (27.6)  858 (70.5)  23 (1.9) | 4.50 | 0.11 |
| Race  *White*  *Non-White* | 2082 (82.8)  425 (16.9) | 1088 (84.1)  205 (15.9) | 994 (81.9)  220 (18.1) | 2.29 | 0.13 |
| Residency  *On campus*  *Off campus* | 385 (15.3)  2128 (84.6) | 181 (14.0)  1114 (86.0) | 204 (16.7)  1014 (83.3) | 3.72 | 0.05 |
| Financial Aid  *Yes*  *No* | 1890 (75.2)  621 (24.7) | 958 (74.0)  336 (26.0) | 932 (76.6)  285 (23.4) | 2.19 | 0.14 |
| Marital status  *Single*  *Partnered* | 1929 (76.7)  584 (23.2) | 971 (75.0)  323 (25.0) | 958 (78.6)  261 (21.4) | **4.44** | **0.04** |
| BMI  *≤ 21.49*  *21.50-23.89*  *23.90-27.49*  $\boldsymbol{\geq}$*27.50* | 607 (26.2)  560 (24.2)  589 (25.5)  558 (24.1) | 321 (27.0)  287 (24.2)  318 (26.8)  261 (22.0) | 286 (25.4)  273 (24.2)  271 (24.0)  297 (26.4) | 6.89 | 0.08 |
| Poor physical health days  0 days  1-3 days  4-9 days  10-30 days  Don’t know | 985 (39.2)  375 (14.9)  355 (14.1)  247 (9.8)  404 (16.1) | 619 (50.8)  193 (15.8)  159 (13.1)  88 (7.2)  159 (13.1) | 366 (30.0)  182 (15.9)  196 (17.1)  159 (13.9)  245 (21.3) | **105.90** | **<0.01** |
| Poor mental health days  0 days  1-3 days  4-9 days  10-30 days  Don’t know | 482 (19.2)  317 (12.6)  439 (17.5)  858 (34.1)  254 (10.1) | 335 (27.8)  193 (16.0)  240 (19.9)  305 (25.3)  134 (11.1) | 147 (12.9)  124 (10.8)  199 (17.4)  553 (48.4)  120 (10.5) | **163.01** | **<0.01** |
| Poor usual activities days  0 days  1-3 days  4-9 days  10-30 days  Don’t know | 893 (35.5)  412 (16.4)  370 (14.7)  396 (15.8)  282 (11.2) | 582 (48.0)  215 (17.7)  180 (14.9)  114 (9.4)  121 (10.0) | 311 (27.3)  197 (17.3)  190 (16.7)  282 (24.7)  161 (14.1) | **158.25** | **<0.01** |
| Age | 24.0 ± 6.20 | 24.40 ± 6.58 | 23.57 ± 5.75 | - | **0.01** |
| Academic Progress Scale | 12.97 ±2.17 | 13.49 ± 1.98 | 12.43 ± 2.23 | - | **<0.01** |
| Student monthly income | 1094.26 ± 1384.27 | 1224.91 ± 1555.67 | 957.51 ± 1163.52 | - | **<0.01** |

α, *p*<0.05, significant values are bolded
