# Supplementary material for: A cross sectional assessment of basic needs insecurity prevalence and associated factors among college students enrolled at a large, public university in the Southeastern U.S
Source: BMC Public Health. 2022 Mar 2;22:419. doi: 10.1186/s12889-022-12817-6 (PMC8889695; doi:10.1186/s12889-022-12817-6)
Supplement: Supplementary file 5 — Additional file 5. Bivariate Analyses of Housing Security Status with Demographic, Financial, and Academic Factors, 2019. Table showing results of chi-square and independent t tests for the demographic, financial, and academic factors verses the variable of interest (housing security status). [file 12889_2022_12817_MOESM5_ESM.docx]

Additional File 5. Bivariate Analyses of Housing Security Status with Demographic, Financial, and Academic Factors, 2019

| Variable | Sample  n (%) | Housing Secure  n (%) | Housing Insecure  n (%) | χ^2^ | p |
| --- | --- | --- | --- | --- | --- |
| Food Security Status  Food Secure  Food Insecure | 1295 (51.5)  1219 (48.5) | 564 (43.6)  287 (23.5) | 731 (56.4)  932 (76.5) | **112.26** | **<0.01** |
| Current Health  *Excellent/Good*  *Fair/Poor* | 1794 (71.4)  585 (23.3) | 661 (82.5)  140 (17.5) | 1133 (71.8)  445 (28.2) | **32.94** | **<0.01** |
| Employed  *Yes*  *No* | 1857 (73.9)  619 (24.6) | 557 (65.6)  281 (33.1) | 1300 (78.2)  338 (20.3) | **49.30** | **<0.01** |
| Ethnicity  *Hispanic*  *Non-Hispanic* | 125 (5.0)  2384 (94.8) | 43 (5.1)  807 (94.9) | 82 (4.9)  1577 (95.1) | 0.02 | 0.90 |
| First Generation  *Yes*  *No* | 609 (24.2)  1904 (75.7) | 182 (21.4)  669 (78.6) | 427 (25.7)  1235 (74.3) | **5.68** | **0.02** |
| Food insecure before college  *Yes*  *No* | 469 (18.7)  2043 (81.3) | 102 (12.0)  748 (88.0) | 367 (22.1)  1295 (77.9) | **37.65** | **<0.01** |
| Family financial support  *Yes*  *No* | 1617 (64.3)  880 (35.0) | 617 (73.5)  222 (26.5) | 1000 (60.3)  658 (39.7) | **42.70** | **<0.01** |
| Year in school  *Sophomore*  *Junior*  *Senior*  *Masters*  *PhD or EdD*  *Professional school* | 479 (19.1)  459 (18.3)  595 (23.7)  468 (18.6)  392 (15.6)  121 (4.8) | 275 (32.3)  155 (18.2)  148 (17.4)  147 (17.3)  92 (10.8)  34 (4.0) | 204 (12.3)  304 (18.3)  447 (26.9)  321 (19.3)  300 (18.0)  87 (5.2) | **162.06** | **<0.01** |
| Gender identity  *Male*  *Female*  *Other* | 720 (28.6)  1754 (69.8)  36 (1.4) | 279 (32.9)  558 (65.7)  12 (1.4) | 441 (26.6)  1196 (72.0)  24 (1.4) | **10.98** | **<0.01** |
| Race  *White*  *Non-White* | 2082 (82.8)  425 (16.9) | 696 (81.9)  154 (18.1) | 1386 (83.6)  271 (16.4) | 1.24 | 0.27 |
| Residency  *On campus*  *Off campus* | 385 (15.3)  2128 (84.6) | 212 (24.9)  639 (75.1) | 173 (10.4)  1489 (89.6) | **91.25** | **<0.01** |
| Financial Aid  *Yes*  *No* | 1890 (75.2)  621 (24.7) | 607 (71.5)  242 (28.5) | 1283 (77.2)  379 (22.8) | **9.81** | **<0.01** |
| Marital status  *Single*  *Partnered* | 1929 (76.7)  584 (23.2) | 697 (82.0)  153 (18.0) | 1232 (74.1)  431 (25.9) | **19.76** | **<0.01** |
| BMI  *≤ 21.49*  *21.50-23.89*  *23.90-27.49*  $\boldsymbol{\geq}$*27.50* | 607 (26.2)  560 (24.2)  589 (25.5)  558 (24.1) | 228 (29.2)  188 (24.1)  192 (24.6)  172 (22.1) | 379 (24.7)  372 (24.3)  397 (25.9)  386 (25.2) | 6.44 | 0.09 |
| Poor physical health days  0 days  1-3 days  4-9 days  10-30 days  Don’t know | 985 (39.2)  375 (14.9)  355 (14.1)  247 (9.8)  404 (16.1) | 398 (49.8)  112 (14.0)  82 (10.3)  69 (8.6)  139 (17.4) | 587 (37.5)  263 (16.8)  273 (17.4)  178 (11.4)  265 (16.9) | **43.83** | **<0.01** |
| Poor mental health days  0 days  1-3 days  4-9 days  10-30 days  Don’t know | 482 (19.2)  317 (12.6)  439 (17.5)  858 (34.1)  254 (10.1) | 205 (25.8)  123 (15.5)  156 (19.6)  222 (27.9)  90 (11.3) | 277 (17.8)  194 (12.5)  283 (18.2)  636 (40.9)  164 (10.6) | **44.90** | **<0.01** |
| Poor usual activities days  0 days  1-3 days  4-9 days  10-30 days  Don’t know | 893 (35.5)  412 (16.4)  370 (14.7)  396 (15.8)  282 (11.2) | 381 (48.0)  136 (17.1)  108 (13.6)  86 (10.8)  83 (10.5) | 512 (32.8)  276 (17.7)  262 (16.8)  310 (19.9)  199 (12.8) | **63.29** | **<0.01** |
| Age | 24.0 ± 6.20 | 24.04 ± 7.39 | 23.98 ± 5.51 | - | 0.83 |
| Academic Progress Scale | 12.97 ±2.17 | 13.24 ± 2.10 | 12.84 ± 2.20 | - | **<0.01** |
| Student monthly income | 1094.26 ± 1384.27 | 1178.81 ±1704.35 | 1052.19 ± 1191.83 | - | **0.03** |

α, *p*<0.05, significant values are bolded
